# COVID-19 Presenting as Acute Bilateral Submassive Pulmonary Embolism in a Young Healthy Female

**DOI:** 10.7759/cureus.9266

**Published:** 2020-07-19

**Authors:** Mina Fransawy Alkomos, Polina Aron, Ian Laxina, Jessimar Sanchez, Michael Agnelli

**Affiliations:** 1 Internal Medicine, St. Joseph's University Medical Center, Paterson, USA

**Keywords:** corona virus disease 2019, pulmonary thromboembolism, computed tomography pulmonary angiography

## Abstract

Similar symptoms, signs, and laboratory abnormalities between coronavirus disease 2019 (COVID-19) and pulmonary embolism (PE) creates a diagnostic challenge to every physician, and emerging data show an association between COVID-19, hypercoagulable state, and venous thromboembolism. We present a rare case of COVID-19 presented as bilateral sub-massive PE. A 28-year-old COVID-19 positive female with no significant past medical history presented with a dry cough and shortness of breath for three days. Initial laboratory test showed elevated D-dimer, electrocardiogram (EKG) showed right axis deviation, right ventricular strain pattern, and S_I_ Q_III_ T_III_ pattern, and echocardiogram (ECHO) showed right ventricular dysfunction. Those two bedside tests directed the urgency of chest CT angiography that showed bilateral sub-massive PE. Since EKG finding of S_I_ Q_III_ T_III_ pattern and right ventricular strain, and ECHO finding of right ventricular dysfunction are well described in PE but not in COVID-19, these bedside diagnostic tools can help identify COVID-19 patients with underlining PEs.

## Introduction

Coronavirus disease 2019 (COVID-19) (caused by the SARS-CoV-2 [severe acute respiratory syndrome coronavirus 2] virus) was first reported in China in December of 2019 [1,2]. Fever, dry cough, fatigue, shortness of breath, pleuritic chest pain, and elevated D-dimer were some of the commonly reported symptoms in two retrospective studies in China; these data overlap with pulmonary embolism (PE) clinical presentation and laboratory tests [1-3]. Electrocardiogram (EKG) and echocardiogram (ECHO) findings are uncommon but well reported in PE; however, they are not well reported in COVID-19 cases [4,5]. Since data is still emerging on COVID-19 associated hypercoagulable status, venous thromboembolism, and PE [6-8], differentiating between COVID-19 and PE based on clinical presentation and laboratory tests alone creates a diagnostic challenge for every clinician.

With the aim of helping to develop a diagnostic algorithm, this case report points out the importance of utilizing EKG and ECHO in guiding the use of CT angiography (CTA) in COVID-19 patients suspected to have a PE. All patient information was de-identified.

## Case presentation

We present the case of a 28-year-old Hispanic female with no significant past medical history who presented to the emergency department (ED) with a worsening dry cough and exertional shortness of breath of three days’ duration. The patient presented to the ED a day prior with the same symptoms, and real-time reverse transcription-polymerase chain reaction assay for SARS-CoV-2 virus was collected, which later turned out to be positive. The patient was vitally stable and was sent home with instructions to return to the ED if the symptoms worsen. On the current presentation, the patient endorses progressive worsening of dry cough and shortness of breath on minimal exertion associated with generalized malaise and left-sided sharp chest pain that is aggravated by cough and deep inspiration. The patient denies fever, nasal congestion, sore throat, hemoptysis, recent travel, lower limb edema, recent surgery, immobilization, trauma, or hormonal therapy. However, the patient reported a family history of provoked deep venous thrombosis in her grandfather secondary to immobilization. On presentation, the patient had a body mass index of 30, was afebrile, and demonstrated normal blood pressure. The patient was hypoxic, saturating 88% on room air that improved to 94% on two liters nasal cannula, had a respiratory rate of 20 breaths per minute, and was also tachycardic at 120 beats per minute. Her physical examination was remarkable for scattered crackles in bilateral lungs, with no lower limb edema or any other acute findings. Laboratory tests were significant for a D-dimer of 17.03 (normal range ≤ 0.50 μg/dL), elevated white blood cell count with neutrophilia and lymphopenia, elevated C-reactive protein, procalcitonin, and interleukin-6, and negative factor V Leiden (Table 1).

**Table 1:** Patient's test results CRP, C-reactive protein; ESR, erythrocyte sedimentation rate; RT-PCR, reverse transcription polymerase chain reaction; SARS-CoV-2, severe acute respiratory syndrome coronavirus 2

Parameters	Normal Range	
White blood cell count, x10^3^/mm^3^	4.5-11	11.2
Neutrophil, x10^3^/mm^3^	1.3-7.8	9.42
Lymphocyte, x10^3^/mm^3^	1-4.6	0.95
Monocyte, x10^3^/mm^3^	0.2-1	0.71
Platelet, K/mm^3^	140-440	148
Prothrombin time, seconds	12.2-14.9	16.6
Activated partial thromboplastin time, seconds	21.3-35.1	31.4
D-dimer, mg/L	≤0.50	17.05
Fibrinogen. mg/dL	183-503	559
Creatine kinase-MB, U/L	30-223	64
Lactate dehydrogenase, U/L	140-271	270
Alanine aminotransferase, U/L	13-39	22
Aspartate aminotransferase U/L	7-52	18
Blood urea nitrogen, mmol/L	7-23	5
Creatinine, mg/dL	0.6-1.3	0.81
Troponin, ng/mL	≤0.03	0.496
Procalcitonin, ng/mL	0.10-0.49	31.64
Ferritin, ng/mL	6.9-283	40
CRP	≤9.9	160.8
ESR, mm/hour	0-20	9
Lactic acid,	0.5-2.2	1.8
Interleukin-6, serum, pg/mL	0-16.4	72.2
Factor V Leiden	Negative	Negative
RT-PCR assay for SARS-CoV-2	Negative	Positive

**Figure 1:**
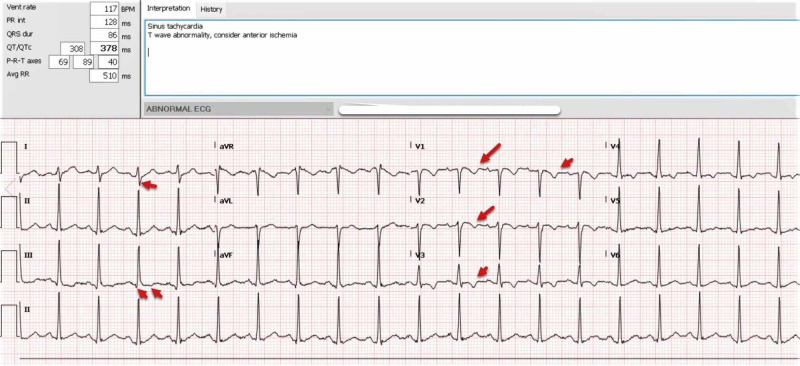
EKG showed sinus tachycardia with right axis deviation, right ventricular strain pattern (T wave inversion in the right pericardial leads V1-V4), and SI QIII TIII

EKG showed sinus tachycardia with right axis deviation, right ventricular strain pattern (T wave inversion in the right pericardial leads V1-V4), and S_I_ Q_III_ T_III_ (Figure 1).

**Figure 2:**
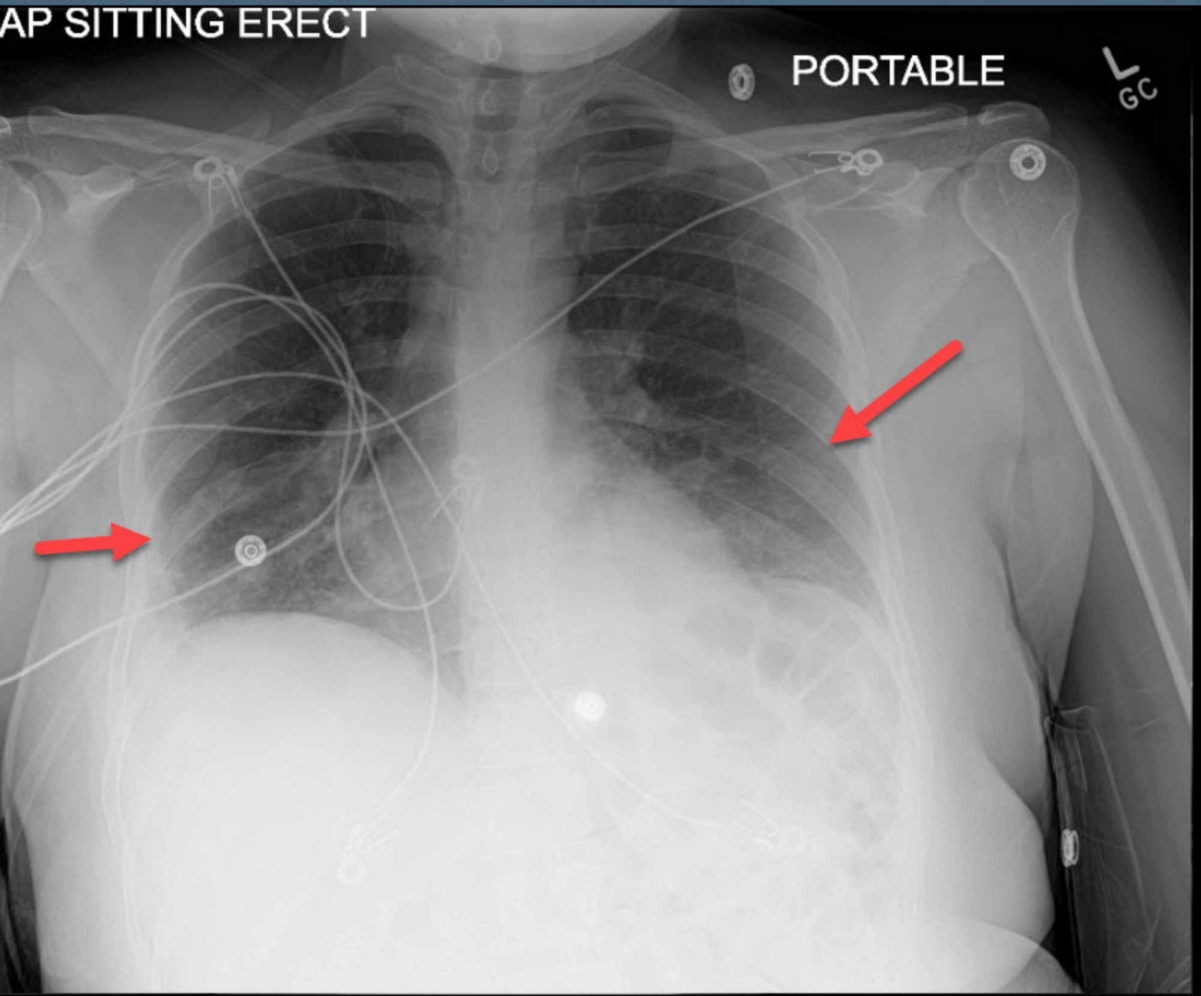
Chest radiography showed scattered consolidation in the left lower lobe and posterior right lower lobe, as well as in regions in the left upper and right upper lobes

Chest radiography showed scattered consolidation in the left lower lobe and posterior right lower lobe, as well as in regions in the left upper and right upper lobes (Figure 2).

**Figure 3:**
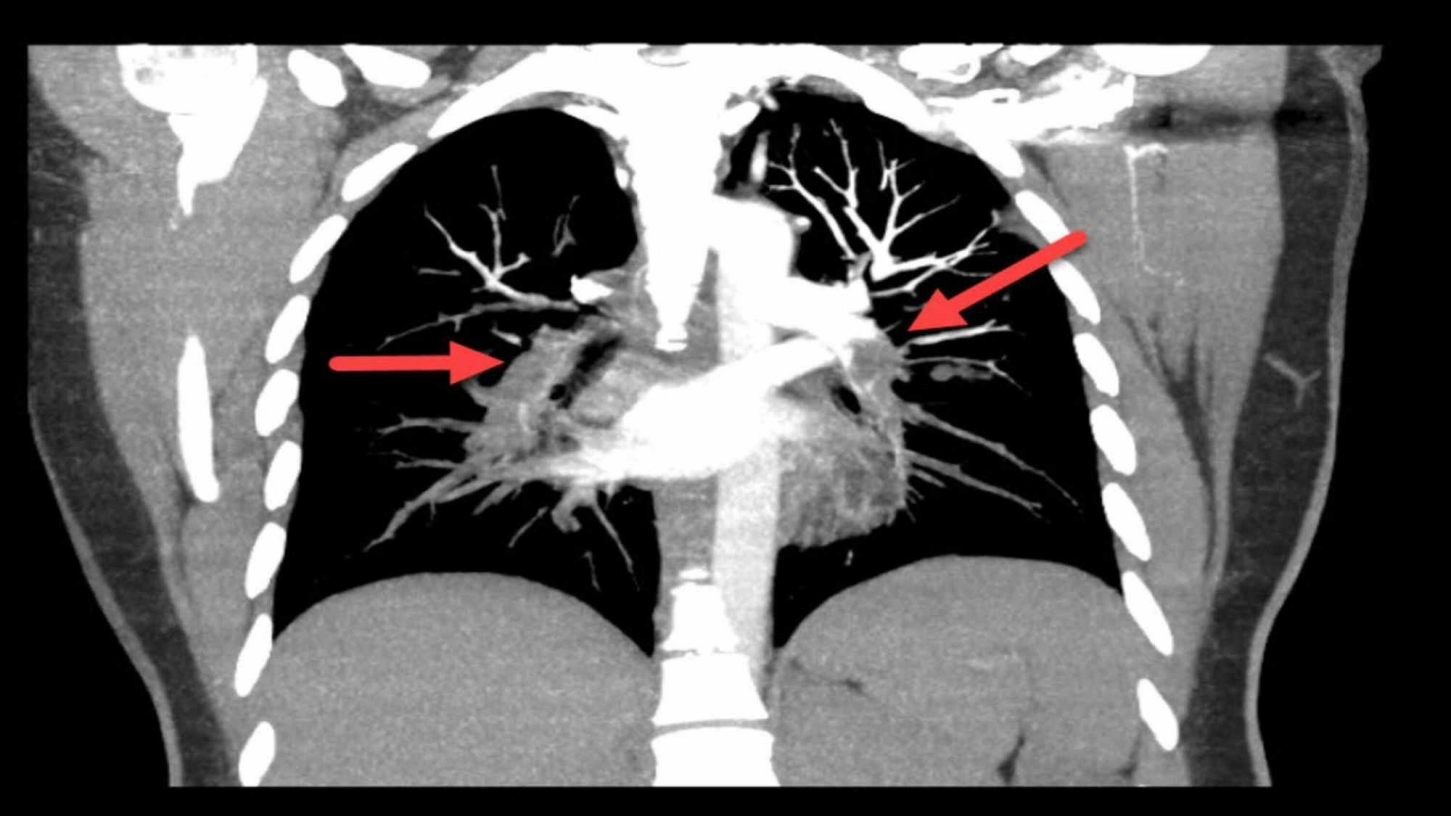
Bilateral pulmonary embolism extending from the main pulmonary arteries into the segmental and peripheral branches

Bedside ECHO showed dilated right ventricle and flattening of the interventricular septum. Based on the EKG, ECHO findings, and the elevated D-dimer, the decision was made to perform CTA of the chest. The test showed bilateral PE extending from the main pulmonary arteries into the segmental and peripheral branches (Figure 3).

The patient was admitted to a negative pressure room, received intravenous tissue plasminogen activator (tPA), and was started on anticoagulation with heparin, azithromycin, and ceftriaxone for coverage of COVID-19 associated pneumonia and community-acquired pneumonia. The patient was happy about the clinical progression of the condition and remained hemodynamically stable. The patient was discharged home after five days on non-vitamin K antagonist oral anticoagulants (NOACs) to be continued for six months.

## Discussion

COVID-19 data are emerging daily. So far, infected patients were reported to have close symptoms of PE, including shortness of breath and pleuritic chest pain, as well as close laboratory abnormalities such as high D-dimer [1-3]. COVID-19 patients were also reported to be at risk of acute kidney injury; therefore CTA in these patients is not encouraged [1,2]. EKG findings in PE are uncommon (less than 10%) but well reported as S_I_ Q_III_ T_III_ pattern, right ventricular strain, and new incomplete right bundle branch block [2]. Also, ECHO findings of right ventricular dysfunction were found in 30-40% out of 3,468 patients diagnosed with PE [9]. Therefore, bedside test as EKG and ECHO can help to identify COVID-19 patients with more likely associated PE.

## Conclusions

Being conscious of the potential overlapping symptoms and laboratory abnormalities between COVID-19 and pulmonary embolism are important for every clinician, as emerging data showed an association between COVID-19, hypercoagulable state, and venous thromboembolism. it will be harmful to perform a CT angiography on every COVID-19 patients as they have a higher risk of acute kidney injury. Therefore, utilizing a bedside diagnostic test like EKG and ECHO can help guide the need for CTA to diagnose PE in COVID-19 patients.
